# Correlation of renin angiotensin and aldosterone system activity with subcutaneous and visceral adiposity: the framingham heart study

**DOI:** 10.1186/1472-6823-12-3

**Published:** 2012-04-04

**Authors:** Conall M O'Seaghdha, Shih-Jen Hwang, Ramachandran S Vasan, Martin G Larson, Udo Hoffmann, Thomas J Wang, Caroline S Fox

**Affiliations:** 1 National Heart, Lung and Blood Institute's Framingham Heart Study, 73 Mt. Wayte Avenue Suite #2, Framingham, MA, USA; 2 Center for Population Studies, Framingham, MA, USA; 3 Renal Division, Brigham and Women's Hospital and Harvard Medical School, Boston, MA, USA; 4 Department of Mathematics and Statistics, Boston University, Boston, USA; 5 Department of Biostatistics, Boston University School of Public Health, Boston, USA; 6 Department of Radiology, Massachusetts General Hospital, Boston, Massachusetts, USA; 7 Cardiology Division, Massachusetts General Hospital, Harvard Medical School, Boston, MA, USA; 8 Division of Endocrinology, Brigham and Women's Hospital and Harvard Medical School, Boston, MA, USA

## Abstract

**Background:**

Animal studies suggest that local adipocyte-mediated activity of the renin-angiotensin-aldosterone system (RAAS) contributes to circulating levels, and may promote the development of obesity-related hypertension in rodents.

**Methods:**

We examined relations of systemic RAAS activity, as assessed by circulating plasma renin activity (PRA), serum aldosterone level, and aldosterone:renin ratio (ARR), with specific regional adiposity measures in a large, community-based sample. Third Generation Framingham Heart Study participants underwent multidetector computed tomography assessment of SAT and VAT volumes during Exam 1 (2002 and 2005). PRA and serum aldosterone were measured after approximately 10 minutes of supine rest; results were log-transformed for analysis. Correlation coefficients between log-transformed RAAS measures and adiposity measurements were calculated, adjusted for age and sex. Partial correlations between log-transformed RAAS measures and adiposity measurements were also calculated, adjusted for standard CVD risk factors.

**Results:**

Overall, 992 women and 897 men were analyzed (mean age 40 years; 7% hypertension; 3% diabetes). No associations were observed with SAT (renin r = 0.04, p = 0.1; aldosterone r = -0.01, p = 0.6) or VAT (renin r = 0.03, p = 0.2; aldosterone r = -0.03, p = 0.2). Similar results were observed for ARR, in sex-stratified analyses, and for BMI and waist circumference. Non-significant partial correlations were also observed in models adjusted for standard cardiovascular risk factors.

**Conclusions:**

Regional adiposity measures were not associated with circulating measures of RAAS activity in this large population-based study. Further studies are required to determine whether adipocyte-derived RAAS components contribute to systemic RAAS activity in humans.

## Background

Obesity, and in particular central obesity, is associated with hypertension, increased cardiovascular risk, diabetes mellitus and chronic kidney disease [1]. Risk estimates from population-based studies indicate that over 65% of hypertension cases may be directly attributable to obesity [2]. Recent increases in the US prevalence of diabetes and chronic kidney disease parallel similar increases in obesity rates, and observational studies demonstrate an association between increasing body mass index (BMI) and these diseases [3-5]. Furthermore, it is proposed that different adipose compartments are associated with varying degrees of metabolic risk [6] and that visceral adipose tissue (VAT) in particular may confer increased risk [7].

The renin-angiotensin-aldosterone system (RAAS) is a cascade of hormones that play a central role in fluid homeostasis and the regulation of vascular resistance, classically activated by the release of renin from the juxta-glomerular apparatus in response to a variety of stimuli, such as renal hypoperfusion [8]. Upregulation of the RAAS has been shown to promote the development of hypertension, hypertension-related end organ damage, atherosclerosis, diabetic end organ damage and chronic kidney disease [9]. Many diverse pathophysiologic mechanisms are implicated, including the production of pro-sclerotic and pro-fibrotic cytokines such as transforming growth factor-β, the promotion of endothelial dysfunction and hypertrophy of vascular beds, the induction of glomerular hypertension [8] and bi-directional interaction with hyperglycemia-induced maladaptive pathways [9].

Adipose tissue, and VAT in particular, may be an important source of non-classical RAAS activity. For example, visceral fat expression of angiotensinogen approximates that of the liver, classically considered the main source of this enzyme [10], and concentrations of angiotensinogen increase with increasing BMI [11]. Non-classical RAAS activation may result from the activity of lipid derived mediators (adipokines) such as complement-C1q TNF-related protein 1 (CTRP1) or adiponectin [12]. For example, CTRP1 is expressed at high levels in adipose tissue and has been shown to induce aldosterone production and partially mediate angiotensin II-induced aldosterone secretion in the obese Zucker rat [12]. In humans, CTRP1 levels are increased in the serum of hypertensive patients, as well as being highly expressed in obese subjects [12]. BMI has also been shown to predict plasma aldosterone concentrations in hypertensive subjects [13], normotensive individuals on a high-salt diet [14], and visceral adiposity is strongly associated with increased blood pressure and hypertension [15].

In light of these observations, we hypothesized that RAAS activity may be related to variation in regional adiposity. To investigate this, we examined relations of RAAS activity, as assessed by circulating plasma renin activity (PRA), serum aldosterone level and aldosterone:renin ratio (ARR), with specific regional adiposity measures in a large, community-based sample. Specifically, we calculated correlations of waist circumference (WC), BMI, VAT and subcutaneous adipose tissue (SAT) with PRA, aldosterone and ARR, and calculated partial correlations adjusted for factors known to be associated RAAS activity.

## Methods

### Study sample

The Framingham Heart Study was initiated in 1948 with the enrollment of 5,209 residents of Framingham, Massachusetts [16]. In 1971, 5,124 offspring of the original cohort participants and their spouses were enrolled, forming the Framingham Offspring Study [17]. Most recently, 4,095 Third Generation Study participants with at least one parent in the Offspring cohort were enrolled between 2002 and 2005 [18]. For the present study, participants were drawn from the 4095 participants who attended the first exam cycle of the Third Generation Study cohort [15]. Exclusion criteria were weight > 350 pounds, pregnancy and age under 35 years for men and under 40 years for women and the presence of CKD (defined as an estimated glomerular filtration rate of < 60 ml/min/1.73^2 ^using the 4-variable MDRD equation [19]). In addition, participants residing in the greater New England area were preferentially included in the CT study for logistical reasons. The institutional review boards of the Boston University School of Medicine and Massachusetts General Hospital approved the study protocol and all subjects provided written informed consent.

### Exposure assessment: Abdominal imaging and measurement of WC, BMI, SAT and VAT

Contemporaneous abdominal imaging was performed with using an 8-slice multidetector CT scanner (Lightspeed Ultra; General Electric, Milwaukee, WI) using a standard protocol [20]. In brief, 25 contiguous 5-mm-thick slices were acquired above the level of S1. To assess SAT and VAT volumes, the abdominal muscular wall separating visceral from subcutaneous fat compartments was manually traced on acquired images and volumetric assessments were made. Intra-reader and inter-reader reproducibility was high, as assessed by 2 independent readers using a subset of 100 randomly selected participants (interclass correlations of 0.997 for SAT and 0.992 for VAT) [20]. These measures have been validated in prior analyses, with differential associations of these VAT and SAT measures previously reported for insulin resistance [21], adiponectin [22], markers of inflammation and oxidative stress [23] and cardiometabolic risk factors [15]. BMI was defined as weight (in kilograms) divided by the square of height (in meters). WC was measured at the level of the umbilicus.

### Outcome assessment: PRA, Aldosterone and ARR

Morning fasting venous blood was drawn after approximately 10 minutes rest in a supine position. Participants were instructed to take all routine medications. Whole blood samples were centrifuged and the serum/plasma fraction stored at -70 to -80°C until analysis. PRA (ng/mL/hr) was measured using the GammaCoat Plasma Renin Activity RIA Kit (DiaSorin). The inter-assay coefficient of variation (CV) was 12.6%. Serum aldosterone (nanograms per deciliter) was measured by radioimmunoassay (Quest Diagnostics) [24]. ARR was calculated by dividing the serum aldosterone level by the PRA.

### Covariate assessment

Covariates were measured at the contemporaneous Framingham Heart Study examination. Fasting plasma glucose, high-density lipoprotein (HDL) cholesterol, and triglycerides were measured on fasting morning samples. Diabetes was defined as a fasting plasma glucose level ≥ 126 mg/dL or treatment with either insulin or a hypoglycemic agent. Current smokers were defined by having smoked at least 1 cigarette per day for the previous year. Alcohol use was assessed by a physician interview and dichotomized on the basis of consumption of more than 14 drinks per week in men or 7 drinks per week in women. Women were considered menopausal if menstruation had ceased for > 1 year. SBP and DBP were measured twice with participant seated after 5 minutes rest and the average of two measures was used for analysis. Hypertension was defined as systolic blood pressure ≥ 140 mm Hg, diastolic blood pressure ≥ 90 mm Hg, or anti-hypertensive treatment.

### Statistical analyses

SAT and VAT were normally distributed, whereas PRA, aldosterone, ARR and triglycerides were all logarithmically transformed to induce approximate normality. Pearson correlation coefficients between continuous adiposity measures (WC, BMI, VAT, SAT) and log-transformed measures of RAAS activity (PRA, aldosterone, ARR) were calculated, adjusting for age and sex. Further associations were evaluated by generating partial correlation coefficients adjusted for standard covariates (age, sex, diabetes, systolic blood pressure, hypertension treatment, HDL cholesterol, log triglycerides and current smoking), as well as covariates known to correlate with radiologic measures of central adiposity (hormone replacement therapy, menopausal state and alcohol use) [25]. Anthropometric measures (BMI or WC) were included in testing correlations with VAT but not for SAT, to avoid co-linearity between SAT and anthropometric indexes [15].

Four secondary analyses were performed. First, linear regression was used to assess the relationship between sex-specific quartiles of VAT and SAT and continuous, log-transformed measures of RAAS activity. Second, we used logistic regression to model the association of continuous VAT and SAT measures with dichotomized measures of RAAS activity, using sex-specific 75^th ^percentile values as cut-offs. Third, we performed a stratified analysis using 3 categories of BMI (lean (BMI < 25), overweight (BMI 25.1 - 29.9) and obese (BMI > 30). Finally, we performed a secondary analysis restricted to participants not taking antihypertensive treatment. All analyses were performed using SAS Version 9.1.3 (SAS Institute), and a two-tailed P value of 0.05 was considered statistically significant. We did not revise P values to account for multiple testing.

## Results

Of the 4095 participants who attended the baseline examination, 1,993 had CT scanning performed with assessment of abdominal SAT and VAT volumes. Of these, 7 were excluded due to the presence of CKD, 11 due to prevalent cardiovascular disease, 36 due to missing adiposity measures, 52 due to missing PRA measurements and 4 due to missing covariates, leaving a final study sample of 1,883 individuals, comprised of 992 women and 897 men. The mean age was 40 years (Table 1); 7% had hypertension and 3% had diabetes. The mean SAT volume was 2729 ± 1331 cm^3 ^and the mean VAT volume was 1592 ± 905 cm^3^.

**Table 1:** Study sample characteristics: Data are means ± SD (normally distributed continuous variables), median (interquartile range) (continuous variables that are not normally distributed), or percent (n)

Clinical measures	
*N*	1889

Age (years)	40 (9)

Women, %	992 (53)

Systolic blood pressure (mmHg)	116 (14)

Hypertension treatment, %	139 (7)

Current smoker, %	323 (17)

Diabetes, %	62 (3)

Postmenopausal, % of women	85 (9)

Hormone Replacement Therapy, % of women	44 (4)

Alcohol use*, %	550/1830 (30)

HDL cholesterol (mg/dl)	54 (16)

Triglycerides (mg/dl)	91 (79, 170)

**Biochemical measures**	

Plasma renin activity (ng/mL/hr)	2.3 (1.1, 2.7)

Serum aldosterone (ng/dL)	12.7 (8.5, 15.0)

**Adiposity measures**	

Body Mass Index (kg/m^2^)	26.6 (5.4)

Overweight (Body Mass Index 25-30 kg/m^2^)	645 (34)

Obese (Body Mass Index > 30 kg/m^2^)	409 (22)

Waist circumference (cm)	96 (13)

Subcutaneous Adipose Tissue (cm^3^)	2729 (1331)

Visceral Adipose Tissue (cm^3^)	1592 (905)

*Defined as > 14 drinks/week

### Correlations With Adiposity Measures

Correlations of SAT, VAT, WC and BMI with log-transformed measures of RAAS activity are shown in Table 2. No associations were observed with SAT (log-renin r = 0.04, p = 0.1; log-aldosterone r = -0.01, p = 0.6), VAT (log-renin r = 0.03, p = 0.2; log-aldosterone r = -0.03, p = 0.2), BMI (log-renin r = 0.03, p = 0.2; log-aldosterone r = -0.04, p = 0.07) or waist circumference (log-renin r = 0.04, p = 0.06; log-aldosterone r = -0.02, p = 0.4) in the overall sample, or in sex-stratified analyses.

**Table 2:** Age-, and sex-adjusted Pearson correlation coefficients (r) of adiposity measures and log-aldosterone to renin ratio, log-aldosterone and log-renin; overall and stratified by sex

	Log-renin		Log-aldosterone		Log-aldosterone: renin	
	**r**	**P-value**	**r**	**P-value**	**r**	**P-value**

**Overall**						

Body Mass Index	0.03	0.2	-0.04	0.07	0.04	0.04

Waist circumference	0.04	0.06	-0.02	0.4	0.05	0.03

Subcutaneous Adipose Tissue	0.04	0.1	-0.01	0.6	0.04	0.1

Visceral Adipose Tissue	0.03	0.2	-0.03	0.2	0.04	0.06

**Men**						

Body Mass Index	0.02	0.6	-0.05	0.2	0.04	0.2

Waist circumference	0.02	0.5	-0.06	0.06	0.05	0.1

Subcutaneous Adipose Tissue	0.01	0.8	-0.04	0.2	0.03	0.4

Visceral Adipose Tissue	0.00	1.0	-0.05	0.1	0.03	0.4

**Women**						

Body Mass Index	0.04	0.2	-0.04	0.2	0.06	0.07

Waist circumference	0.06	0.05	0.02	0.6	0.04	0.2

Subcutaneous Adipose Tissue	0.06	0.06	0.01	0.6	0.04	0.2

Visceral Adipose Tissue	0.06	0.05	-0.01	0.8	0.06	0.07

### Partial correlation with SAT, VAT, and Metabolic Risk Factor Variables

Results of partial correlation tests for SAT, VAT, WC and BMI are shown in Table 3. No significant association between log-renin and SAT (p = 0.1), VAT (p = 0.2), BMI (p = 0.7) or WC (p = 0.06) was observed. Also, no significant associations were observed for log-aldosterone and SAT (p = 0.7), VAT (p = 0.2), BMI (p = 0.8) or WC (p = 0.4). Additional adjustment for specific adiposity measures (BMI or VAT) did not significantly affect the results. We determined that we had 80% power to detect a correlation of 0.065, if it truly existed.

**Table 3:** Partial correlation coefficients (r) between adiposity measures and log-aldosterone to renin ratio, log-aldosterone and log-renin, adjusted for cardiovascular risk factors

	Log-renin		Log-aldosterone		Log-aldosterone: renin ratio	
	**r**	**P-value**	**r**	**P-value**	**r**	**P-value**

**SAT**						

Multivariable-adjusted	0.03	0.1	-0.01	0.7	0.04	0.1

**VAT**						

Multivariable-adjusted	0.03	0.2	-0.03	0.2	0.04	0.05

Multivariable-adjusted + BMI	0.03	0.1	-0.03	0.2	0.05	0.04

Multivariable-adjusted + SAT	0.02	0.4	-0.03	0.2	0.03	0.1

**BMI**						

Multivariable-adjusted	0.01	0.7	-0.01	0.8	0.01	0.6

Multivariable-adjusted + VAT	0.01	0.8	-0.01	0.6	0.01	0.6

**Waist circumference**						

Multivariable-adjusted	0.04	0.06	-0.02	0.4	0.05	0.04

Multivariable-adjusted + VAT	0.03	0.2	0.01	0.7	0.02	0.4

*Multivariable-adjusted for age, sex, diabetes, systolic blood pressure, hypertension treatment, HDL-cholesterol, log triglycerides, menopause status, hormone replacement therapy and current smoking.

### Secondary analyses

Following the negative results of the primary analysis, we tested the association of quartiles of VAT and SAT and continuous RAAS measures in linear regression models. No significant association was observed for either VAT (multivariable-adjusted relations of uppermost vs. lowermost quartile and log aldosterone concentration: p = 0.2) or SAT (multivariable-adjusted relations of uppermost vs. lowermost quartile and log-aldosterone concentration p = 0.7) in this analysis.

In a logistic regression model of continuous SAT and VAT measures with dichotomized measures of RAAS activity (using the 75^th ^percentile as a cut-point), no significant association was observed for either SAT or VAT and plasma renin level (multivariable-adjusted odds ratio [OR] of the uppermost quartile of renin concentration per standard deviation increment in SAT was 1.10 (95% confidence interval [CI] 0.99 - 1.22; p-value = 0.07) and VAT was 1.09 (95% CI 0.99 - 1.21; p-value = 0.09) or plasma aldosterone level (OR per standard deviation increase in SAT was 0.93 (95% CI 0.84 - 1.02; p-value = 0.2) and VAT was 0.92 (95% CI 0.84 - 1.02; p-value = 0.1).

In a stratified analyses using sub-categories of BMI (lean (BMI < 25), overweight (BMI 25.1 - 29.9) and obese (BMI > 30), we observed a weak correlation between renin activity and renin: aldosterone ratio and SAT in lean individuals (r for plasma renin and SAT 0.08; p-value 0.03; r for renin: aldosterone ratio and SAT 0.08; p-value 0.03). There were no significant correlations apart from this, similar to the primary analysis.

Finally, apart from a weak association between renin: aldosterone ratio and BMI (r = 0.05; p-value 0.04), results of analyses undertaken in participants not taking antihypertensive treatment were essentially unchanged from the primary analysis, with no significant correlations observed.

## Discussion

Contrary to our original hypothesis, we did not observe an association between any specific adiposity measure and activity of the RAAS system in overall, sex-stratified or multivariable-adjusted analyses.

Angiotensinogen, renin (or peptides with renin-like activity), angiotensin converting enzyme (ACE), and AT_1 _and AT_2 _receptors are all secretory products of the rodent adipocyte [26], and the expression of some are increased in obesity [27,28]. Although their primary function is not known, autocrine/paracrine tissue effects on adipocyte function are well described [29,30]. In addition, studies in transgenic mice demonstrate that adipose-derived angiotensinogen may also contribute to the systemic RAAS pool, and the relationship between obesity and hypertension may be directly explained by angiotensinogen secretion by the rodent adipocyte [31].

As in rodents, multiple lines of evidence support the existence of adipocyte RAAS in humans [30], and the present findings do not preclude the existence of significant local RAAS activity in our sample. However, evidence that the human adipose RAAS makes a significant contribution to systemic activity has not been convincingly demonstrated, and the literature in this area is conflicting. For example, a study in lean, healthy, young men (n = 91) did demonstrate a correlation between plasma angiotensinogen levels and BMI (r = 0.33, P < 0.05), whereas no significant correlation with PRA or circulating aldosterone levels was observed [11]. In a second study of 38 obese vs. lean postmenopausal women, circulating levels of angiotensinogen, renin, aldosterone and angiotensinogen converting enzyme (ACE) were significantly higher in the obese women, and plasma levels of several RAAS peptides decreased after weight loss [32]. However, in contrast to our population-based study, morbidly obese cases were selected for this analysis (mean BMI 38 kg/m^2^), making direct comparison with our study difficult. It is possible that unaccounted factors may have influenced RAAS levels in that study; for example, there was evidence of substantial insulin resistance in cases, which has been shown to be independently associated with RAAS activity in many studies [33-37]. A third study also detected a correlation between BMI and plasma aldosterone concentrations in overweight and obese patients with essential hypertension [13]. However, this was conducted in a tertiary referral hypertension clinic, and is not directly comparable with the present analysis. Furthermore, several studies in obese individuals with hypertension have not detected an association between obesity and circulating RAAS components [38-40].

The present work extends the literature in several ways. In contrast to evidence from animal models, where adipocyte RAAS exerts substantial physiologic action beyond the local environment of the adipocyte [29], our findings suggest that adipose RAAS does not make a substantial contribution to the systemic RAAS pool in the general population. There are several potential reasons for this. First, substantial inter-species differences in fat deposits and metabolism exist between rodents and humans [41]. For example, whereas angiotensin II promotes adipocyte growth and pre-adipocyte recruitment in rodents, it is anti-adipogenic in human adipose tissue [41]. Furthermore, in contrast to rodent models of diet-induced obesity, angiotensinogen mRNA expression in the adipose tissue of obese, hypertensive females is not greater than that of non-obese controls [30]. Fundamental inter-species differences in adipocyte biology such as these may preclude the extension of observations made in the rodent to humans. Equally, even if observations made in obese rodents are reflective of human pathophysiology, the cross-sectional design of our study may fail to capture dynamic changes in RAAS components due to active weight gain. For example, changes in the expression of RAAS components following sudden, controlled weight gain in rodents may not be observed during chronic, stable obesity, which may be more representative of the participants in our study. As discussed, significant changes in circulating RAAS components have been demonstrated in response to acute weight loss in humans [32]. Finally, it may be that adipose RAAS functions primarily in a paracrine/autocrine manner, and systemic levels are not reflective of local tissue activity in humans.

Several avenues for further study are suggested by the present analysis. In particular, efforts to delineate the relative importance of adipocyte-derived (as compared to classically-derived) RAAS components, and their relative contribution to circulating levels, are required; adipocyte-specific RAAS component knockout models would be most helpful in this regard. Human studies comparing tissue mRNA expression of RAAS components during weight gain as compared to chronic obesity and lean controls may also prove illuminating.

The use of an unselected, community-based sample, the highly reproducible volumetric method of assessing SAT and VAT, and the sample size sufficient to power multivariable analyses comparing the relative magnitudes of association strengthen our study. However, several limitations should also be acknowledged. Ambulatory RAAS measurements were drawn from individuals on a free-living sodium diet, while taking their usual antihypertensive medications, potentially biasing our results toward the null. As sodium balance is a major determinant of renin and aldosterone, the absence of data on sodium intake and urinary excretion is an important limitation. However, it should be noted that results were similar to the primary analysis in participants not taking anti-hypertensive treatment. Furthermore, samples were drawn after only 10 minutes in the supine position, and not after 1 h of rest as recommended in research unit protocols, which are impractical in the setting of a large, longitudinal observational cohort. Our analysis would be enhanced by directly measuring markers of local RAAS activation in adipose tissue. However, this is unfortunately not technically feasible in a large, observational epidemiologic study. Finally, our results may not be generalizable to all racial/ethnic groups or age groups, as our sample was primarily white and middle-aged, and racial differences in serum aldosterone and renin values have been reported [42].

## Conclusions

In conclusion, regional adiposity measures were not associated with systemic RAAS activity, as determined by plasma renin activity, serum aldosterone concentration and aldosterone to renin ratio, in this large population-based study. Further studies are required to establish whether adipose-derived RAAS components contribute to systemic activity in humans.

## Competing interests

The authors declare that they have no competing interests.

## Authors' contributions

COS contributed to the conception and design of this study, interpretation of data, and drafting the manuscript. SJH and MGL contributed to the analysis and interpretation of data and revising the article critically for important intellectual content. RSV and UH revised the manuscript critically for important intellectual content. CSF and TJW contributed to the conception and design of this study, analysis and interpretation of data, and drafting the manuscript and revising the manuscript for important intellectual content. All authors read and approved the final manuscript.

## Pre-publication history

The pre-publication history for this paper can be accessed here:

http://www.biomedcentral.com/1472-6823/12/3/prepub
